# Identifying candidate sites for crop biofortification in Latin America: case studies in Colombia, Nicaragua and Bolivia

**DOI:** 10.1186/1476-072X-8-29

**Published:** 2009-05-19

**Authors:** Emmanuel Zapata-Caldas, Glenn Hyman, Helena Pachón, Fredy Alexander Monserrate, Liliana Vesga Varela

**Affiliations:** 1 Department of Geography, Universidad del Valle, Cali, Colombia; 2 International Center for Tropical Agriculture (CIAT), Cali, Colombia; 3 Universidad Industrial de Santander, Bucaramanga, Colombia

## Abstract

**Background:**

Agricultural science can address a population's vitamin, amino acid and mineral malnutrition through biofortification - agronomy, plant breeding and biotechnology to develop crops with high nutrient contents. Biofortified crop varieties should be grown in areas with populations at risk of nutrient deficiency and in areas where the same crop is already grown and consumed. Information on the population at risk of nutrient deficiency is rarely available for sub-national administrative units, such as provinces, districts, and municipalities. Nor is this type of information commonly analyzed with data on agricultural production. This project developed a method to identify populations at risk of nutrient deficiency in zones with high crop production, places where biofortification interventions could be targeted.

**Results:**

Nutrient deficiency risk data were combined with crop production and socioeconomic data to assess the suitability of establishing an intervention. Our analysis developed maps of candidate sites for biofortification interventions for nine countries in Latin America and the Caribbean. Results for Colombia, Nicaragua, and Bolivia are presented in this paper. Interventions in northern Colombia appear promising for all crops, while sites for bean biofortification are widely scattered throughout the country. The most promising sites in Nicaragua are found in the center-north region. Candidate sites for biofortification in Bolivia are found in the central part of the country, in the Andes Mountains. The availability and resolution of data limits the analysis. Some areas show opportunities for biofortification of several crops, taking advantage of their spatial coincidence. Results from this analysis should be confirmed by experts or through field visits.

**Conclusion:**

This study demonstrates a method for identifying candidate sites for biofortification interventions. The method evaluates populations at risk of nutrient deficiencies for sub-national administrative regions, and provides a reasonable alternative to more costly, information-intensive approaches.

## Background

The most common strategies to address vitamin, amino acid and mineral deficiencies are diet diversification, supplementation and fortification. A relatively new strategy – biofortification – is the improvement of agronomic characteristics and the nutritional content of crops through agronomy, plant breeding and biotechnology [1]. Biofortification can be achieved through agronomic or genetic approaches [2].

Agronomic strategies to biofortify crops include fertilizer and soil management to increase the amount of nutrients available for absorption by the plant. While these soil management strategies have proved successful in many cases [2], the present study seeks to identify locations where new crop varieties, developed through plant breeding or biotechnology, can be grown in areas with populations at risk of micronutrient deficiency. Global or regional initiatives to develop soil management strategies for biofortification have not yet been widely implemented. Moreover, populations suffering from micronutrient deficiencies are less likely to be able to afford fertilizers needed to implement soil management strategies for biofortification. For these reasons, this paper and our use of the term "biofortification" refer to plant breeding or biotechnology, to improve the nutrient content of crop varieties.

Biofortification through plant breeding takes advantage of the natural genetic diversity of crops, genetically crossing different varieties of a crop to develop new cultivars with higher levels of desired nutrients. These new varieties can be disseminated to farmers in areas where nutrient-dense crops could address problems of nutrient deficiency and malnutrition. Several studies have shown that biofortification can improve nutritional status and that it is economically viable [3-6]. Major international programs [7,8] have been initiated to breed crops with higher levels of iron, zinc, Vitamin A, and the precursors to protein tryptophan and lysine (hereafter referred to as "amino acid"). These nutrients are also the focus of this paper.

Biofortified crop varieties should be disseminated and used in places where nutrient deficiency is a problem and where the crops of interest are being produced and consumed in sufficient quantity to achieve impact. If these conditions are not met, then investments in biofortified crops will fail to reach the intended beneficiaries. A growing body of research has demonstrated the benefits of geographic targeting for poverty reduction and improving nutrition [9-11]. Thus, the targeting of interventions is an important problem that any nutritional initiative must address.

Our analysis combines agricultural, nutritional, and socioeconomic information to assess candidate sites for iron, zinc, vitamin A and amino acid biofortification in nine countries of Latin America and the Caribbean. Results for three of the countries are presented here. Results for the remaining countries can be accessed on the Web Site:*Identificación de Sitios Candidatos para la Biofortificación de Cultivos en Latinoamérica y El Caribe *[12]. Candidate sites for biofortification interventions are found in areas where high prevalence of nutritional risks, high production and consumption of target staple crops, and high risk of poverty converge. This analysis is a preliminary step, before more detailed research on the candidate sites can determine their potential for impact.

## Methods

The analysis first employed a procedure to prioritize indicators of nutrient deficiency risk. Next, weighted overlay was used to generate scores indicating the degree of confluence of factors important for biofortification. Data were collected to reflect the demand for nutrition interventions and the presence of the staple crops that are the current focus of biofortification research to improve nutrient content. The method assigned scores to the collected variables that are relevant to targeting biofortification interventions. The variables were weighted according to their importance to the result. The scores were then summed at the pixel level to provide the final result map. The following sections describe the data collected and the weighted overlay procedure.

### Data on risk of nutrient deficiency

**Figure 1:**
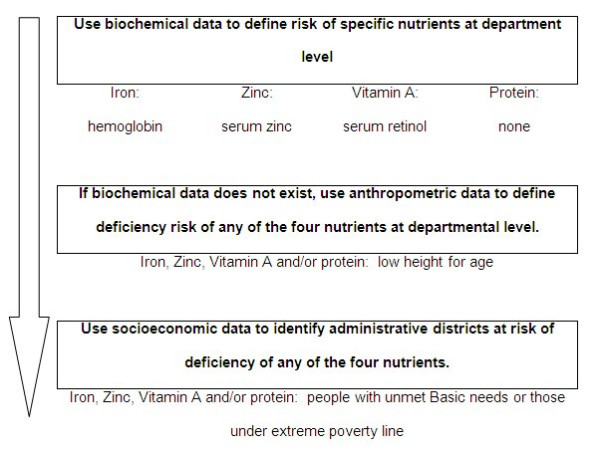
**Schema for selecting nutrient deficiency risk indicators**.

Assessing the demand for nutrition interventions over a large region calls for the development of information characterizing the magnitude and geographic distribution of nutrient deficiencies. A literature review of indicators of nutrient deficiency was carried out to determine the most appropriate indicators and how they could be used. Our assessment of the literature suggested a hierarchical organization of nutrient risk indicators based on how well they depict the problem. Indicators were grouped into three categories – biochemical measures, anthropometric measurements of children, and socioeconomic status (Figure 1). The measures were then classified according to the literature review into risk levels of nutrient deficiency. The class breaks and assignments of scores for the weighted overlay method are consistent with the scientific literature regarding risk levels of the three categories of nutritional indicators. The indicators of nutritional risk were linked to administrative division maps and analyzed in a geographic information system.

The type of nutrient risk indicator used in the analysis was determined according to usefulness first, and then according to data availability. Biochemical measures of nutrient deficiency, such as hemoglobin levels in the blood, are direct measures of a person's nutrient status, and as such are the preferred indicator. Unfortunately, health surveys often lack such data, especially for measures of Vitamin A and levels of amino acids. When biochemical indicators were unavailable, anthropometric indicators were the preferred next option. Since national health surveys often include anthropometric measurements of children less than 5 years of age, this indicator is often available [13]. Finally, if neither biochemical nor anthropometric data are available, poverty measures and maps can serve as indicators of risk of nutrient deficiency.

### Data on population and poverty

Biofortification interventions are more likely to be successful where there are substantial rural populations living in poverty. Rural population data were developed from the Gridded Population of the World data set [14]. The 1-km global data set was resampled to 10-km resolution to conform to the framework of the analysis. Poverty index maps were derived from vector maps at the 2^nd ^administrative level for Latin America based on the basic needs method [15]. These maps were converted to raster format.

### Data on crop production and consumption

Biofortification interventions necessarily must be implemented where farmers grow the crop and consumers provide a local market. Several measures of the presence of the target crop for biofortification were collected and mapped. Biofortification is more likely to have a nutritional impact where there is a high level of production and consumption of the crop. Crop data sets for this analysis were derived from 10-km resolution crop production maps available for the world [16,17]. Food consumption data were acquired at department level from the Living Standards Measurement Study [18]. FAO production statistics and food balance sheet data provided contextual information for the analysis [19].

### Weighted overlay analysis

The first step in carrying out a weighted overlay analysis was to convert input data to the same spatial format and framework. A raster format was developed with 10-km spatial resolution to match the crop production data. All vector maps were converted to raster formats with corresponding 10-km pixel resolution. The literature review mentioned above had classified risk of nutrient deficiency into low, moderate, and high, and in some cases added an additional category of very high. Values of 3 (low), 6 (moderate), and 9 (high) were assigned when the classification comprised three categories. Values of 3 (low), 5 (moderate), 7 (high), and 9 (very high) were assigned when the classification included four categories. All other data were divided into terciles and assigned three values depending on whether they fell into the lowest (3), middle (6), or highest (9) tercile.

The next step was to assign influence weights to each variable according to the importance of that variable to biofortification interventions. The risk of nutrient deficiency and the presence of crop production were considered to be the most important factors, and each was assigned an influence weight of 30%. Poverty intensity and rural population density were both assigned influence weights of 20%, since the weights must add up to 100%. The weighting scheme can be altered in the future, after dialogue with country experts on the preliminary results presented thus far.

**Figure 2:**
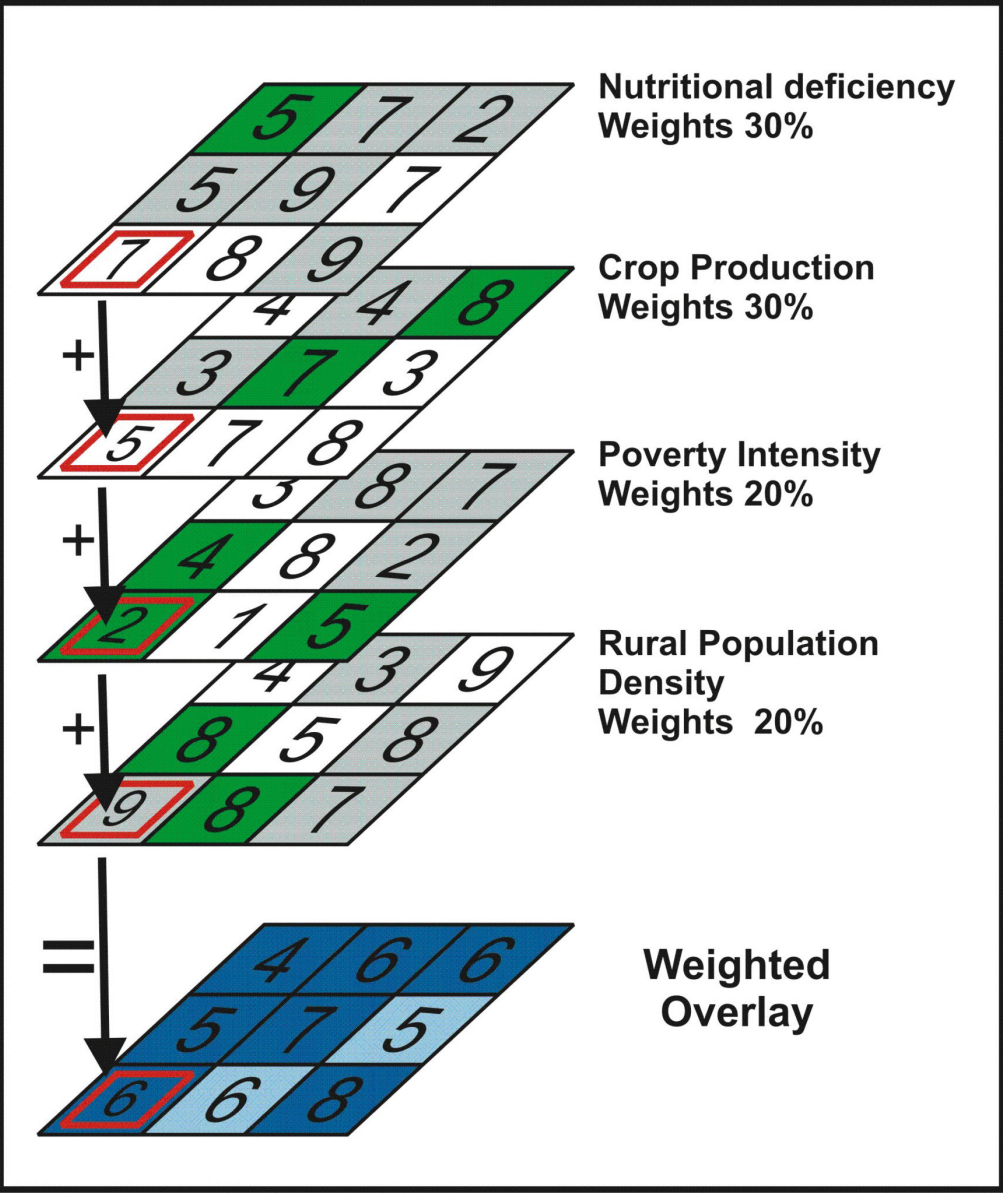
**Use of the weighted overlay method for lower left pixel in a hypothetical map**. Adapted from ESRI, 2006 [20].



Figure 2 illustrates the method for the lower left pixel in a hypothetical map [20]. For each pixel, the assigned influence weights were multiplied by the corresponding variable value and then summed to derive the final score:

Where *a *is the indicator of nutrient deficiency risk, *b *is the level of crop production, *c *is the poverty intensity, and *d *is the rural population density. The example in Figure 2 shows a high value of 7 for nutrient deficiency risk and a moderate value of 5 for crop production. With a value of 2, poverty intensity is low for the pixel. A rural population density value of 9 is high. Applying these values to the equation above yields a final score for the pixel of 6 (scores are rounded to the nearest integer).

The map resulting from this weighted overlay procedure shows high, moderate, or low scores depending on the confluence of factors relevant to biofortification interventions. The highest scores indicate areas where the combinations of factors suggest a candidate site for implementing a biofortification program. The maps were further improved by eliminating isolated pixels surrounded by non-similar values through application of a spatial filter to the data. Finally, the highest two scores were chosen for the final map.

## Results

### Colombia

**Figure 3:**
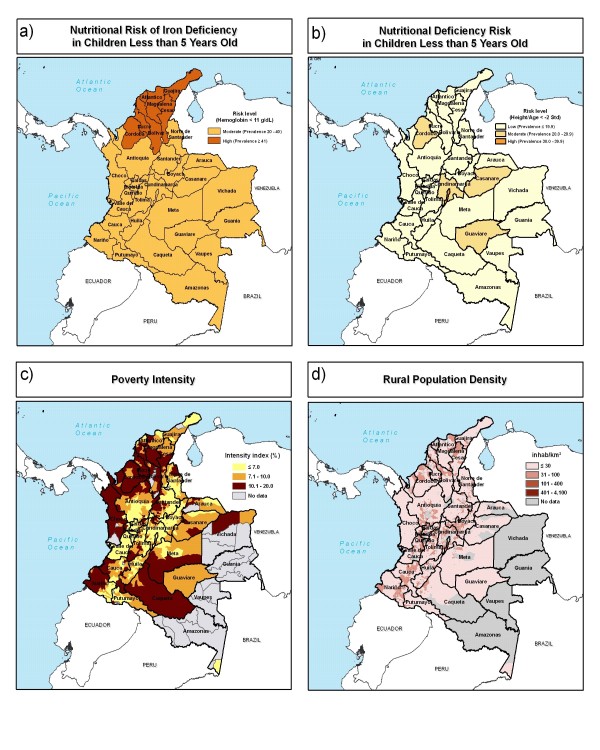
**The geographic distribution of rural population, poverty intensity, and risks of nutrient deficiency in Colombia**. a) hemoglobin levels and b) stunting (height for age) in children less than 5 years of age, c) poverty intensity, and d) rural population density. Sources: ICBF, 2005 [21] (iron deficiency); MACRO International, 2007 [13] (nutrient deficiency); Schnuschny and Gallopin, 2004 [15] (poverty intensity); CIESIN et al., 2004 [14] (rural population density).

Biofortification interventions in Colombia could potentially be implemented in any of the four physiographic regions – the coast, mountains, savanna (Llanos), and Amazon (Figure 3). Population density is highest in the inter-Andean valleys of the mountain regions, areas such as the Bogotá plain (Cundinamarca) and the Cauca Valley. The savanna, Amazon, and coastal regions have far fewer people, but higher proportions of their population living in poverty (Figure 3c and 3d).

All departments in Colombia have either moderate or high risks of iron deficiency as indicated by hemoglobin levels surveyed in the Demographic and Health Survey [21] (Figure 3a). A group of departments in the north has high risks of iron deficiency. The map of stunted children shows a group of four departments with moderate levels of nutrient deficiency risk (Figure 3b). The federal district has high risk of nutrient deficiency as indicated by stunted children.

**Figure 4:**
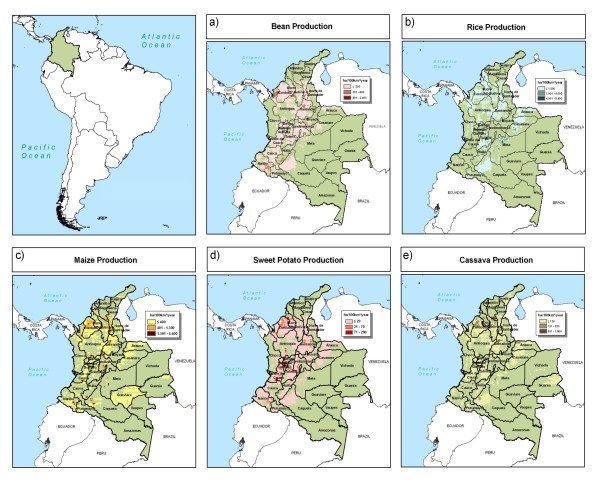
**Crop production in Colombia**. a) bean, b) rice, c) maize, d) sweet potato, and e) cassava. Source: You and Wood, 2006 [16].

Colombian crops that are the focus of biofortification efforts are found mainly in the hills and valleys of the mountain region (Figure 4). Nariño, Santander, and Antioquia are important regions for beans. Cassava production is most dense in the northern part of the country. Key areas of rice production include the Llanos (Meta department), the Amazon regions bordering the Andes Mountains, and many coastal regions in the northern part of the country. Maize has a fairly wide distribution throughout the country, with high production in Antioquia and Córdoba.

**Figure 5:**
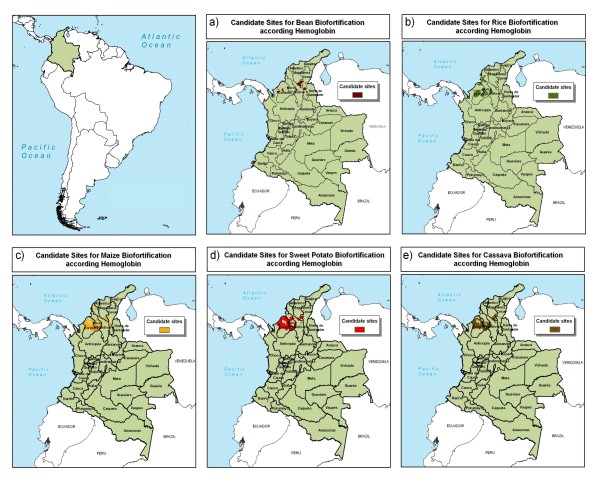
**Candidate sites for iron biofortification in Colombia**. a) bean, b) rice, c) maize, d) sweet potato, and e) cassava, as indicated by hemoglobin levels. Source: AgroSalud, 2007 [8].

High anemia levels in northern Colombia suggest this area as a best bet for candidate sites to implement crop biofortification aimed at reducing iron deficiency (Figure 5). In particular, the Córdoba department could be a focus for improved cassava, sweet potato, maize, and rice. High scores also were found in the southern parts of both Magdalena and Sucre departments. The result map indicates potential sites for bean biofortification in the northern part of the country and some smaller areas scattered throughout the country.

**Figure 6:**
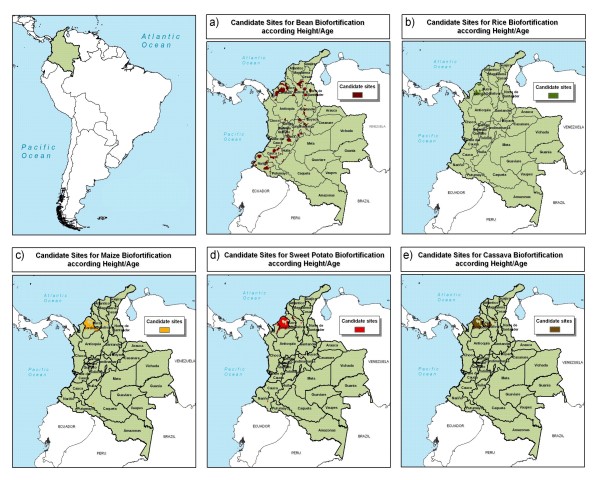
**Candidate sites for zinc, amino acids, and vitamin A biofortification in Colombia**. a) bean, b) rice, c) maize, d) sweet potato, and e) cassava, as indicated by height-for-age. Source: AgroSalud, 2007 [8].

Candidate sites for biofortification with zinc, amino acids, and/or vitamin A are similar to those for iron (Figure 6). The Córdoba department in northern Colombia could be a focus of intervention for all crops. One exception to the focus on the northern part of the country is the pattern for bean biofortification, where pockets of bean production throughout the Andes coincide with moderate levels of stunting or high poverty intensity.

### Nicaragua

**Figure 7:**
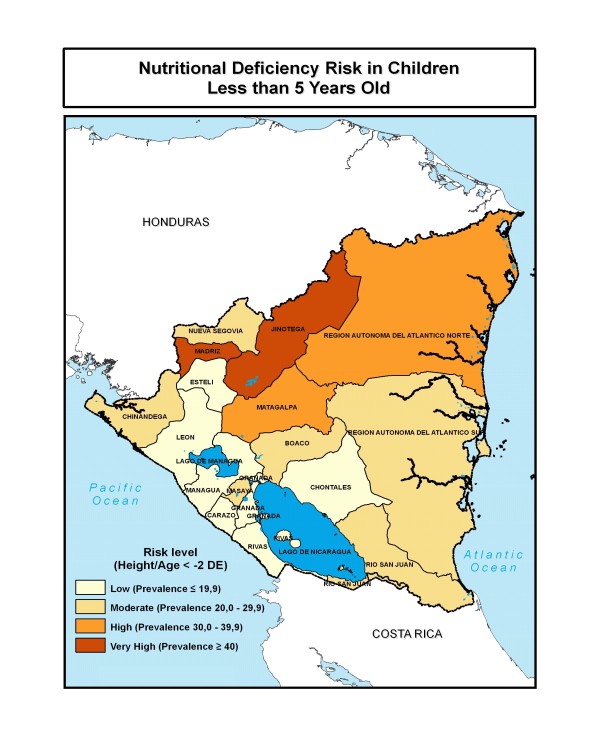
**Nutritional risk of zinc, vitamin A, and amino acids deficiency in Nicaragua, as indicated by height-for-age**. Source: INEC, 2002 [32].

Only general deficiency risk, based on anthropometry, could be evaluated for Nicaragua because of the lack of biochemical data on specific nutrients (Figure 7). High and very high risk levels are found in the northern departments. Moderate risks are found in the southeast part of the country, with low risks in the east.

**Figure 8:**
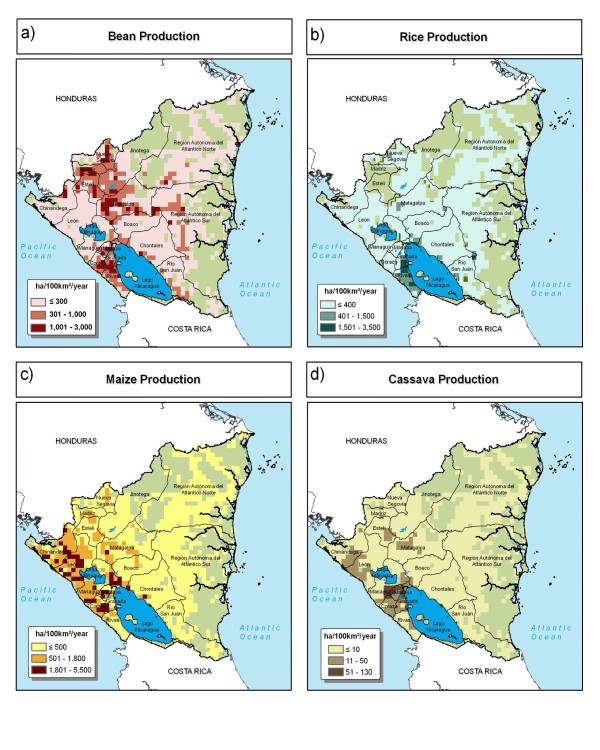
**Crop production in Nicaragua**. a) bean, b) rice, c) maize, and d) cassava. Source: You and Wood, 2006 [16].

Crop production is mostly focused in the western part of Nicaragua (Figure 8). Much of the humid east lacks large-scale production. Maize cultivation is concentrated in the departments along the Pacific Ocean. Bean production is most dense to the west of Lake Nicaragua and a group of departments in the center-north region.

**Figure 9:**
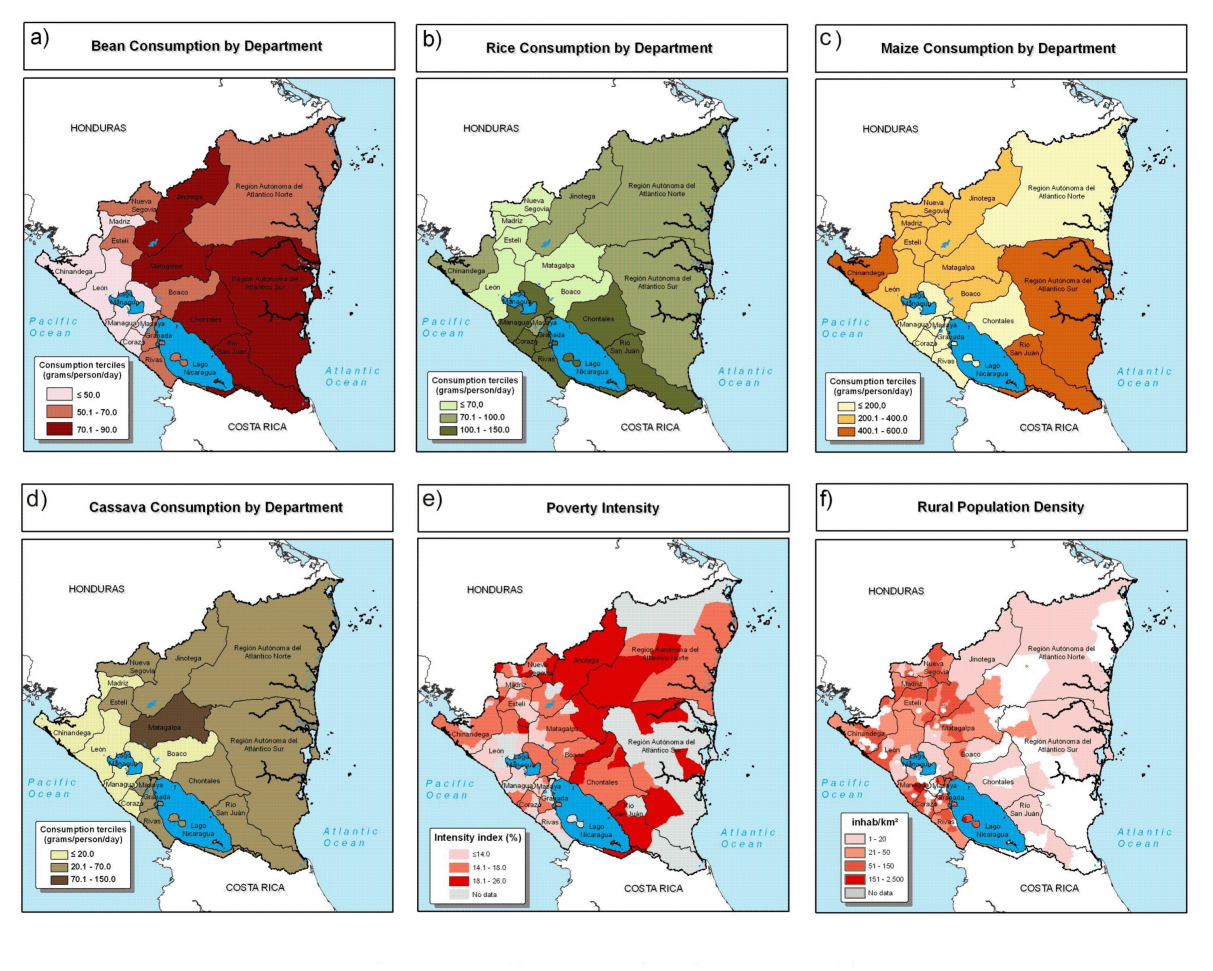
**Food crop consumption, poverty, and population in Nicaragua**. a) bean, b) rice, c) maize, and d) cassava consumption by department, e) poverty intensity, and f) rural population density. Sources: World Bank, 2008 [18] (consumption); Schuschny and Gallopin, 2004 [15] (poverty intensity); and CIESIN et al., 2004 [14] (rural population density).

Consumption of beans, rice, and maize generally follows production patterns (Figure 9). The exceptions are Río San Juan and Atlántico Sur departments where per capita consumption is high. However, these departments have relatively low population density.

Nicaraguans consume large quantities of maize and beans, moderate quantities of rice, and modest amounts of cassava or sweet potato. Nicaragua neither imports nor exports large volumes of maize, beans, rice, and cassava [19]. Thus, consumption of biofortified varieties of these crops – mostly grown within the country – would be likely to reach the intended beneficiaries.

**Figure 10:**
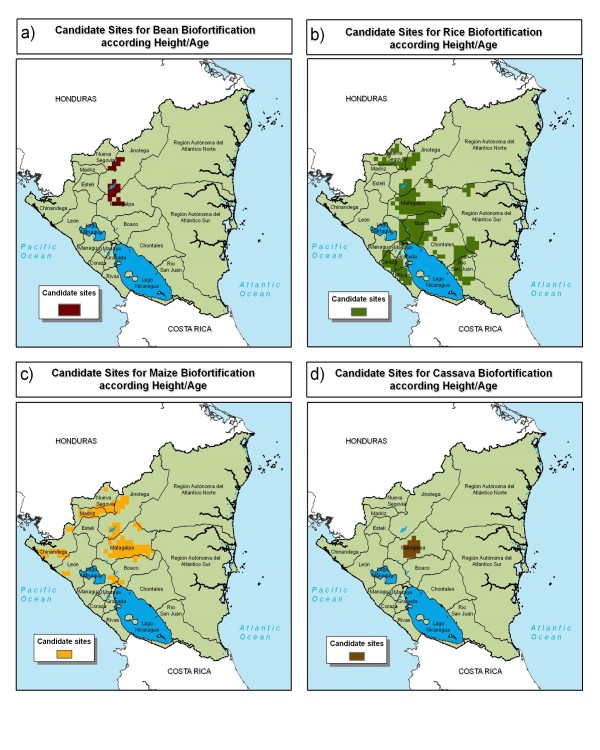
**Candidate sites for zinc, amino acids, and vitamin A biofortification in Nicaragua**. a) bean, b) rice, c) maize, and d) cassava as indicated by height-for-age. Source: AgroSalud, 2007 [8].

The center-north region stands out as a likely candidate for biofortification interventions (Figure 10). Matagalpa department shows candidate sites for bean, rice, maize, and cassava. Jinotega department shows candidate sites for rice, maize, and bean. Bean and cassava candidate sites are concentrated in relatively small areas in the center-north of the country. Maize and rice candidate sites are distributed widely, following production zones of these crops.

### Bolivia

**Figure 11:**
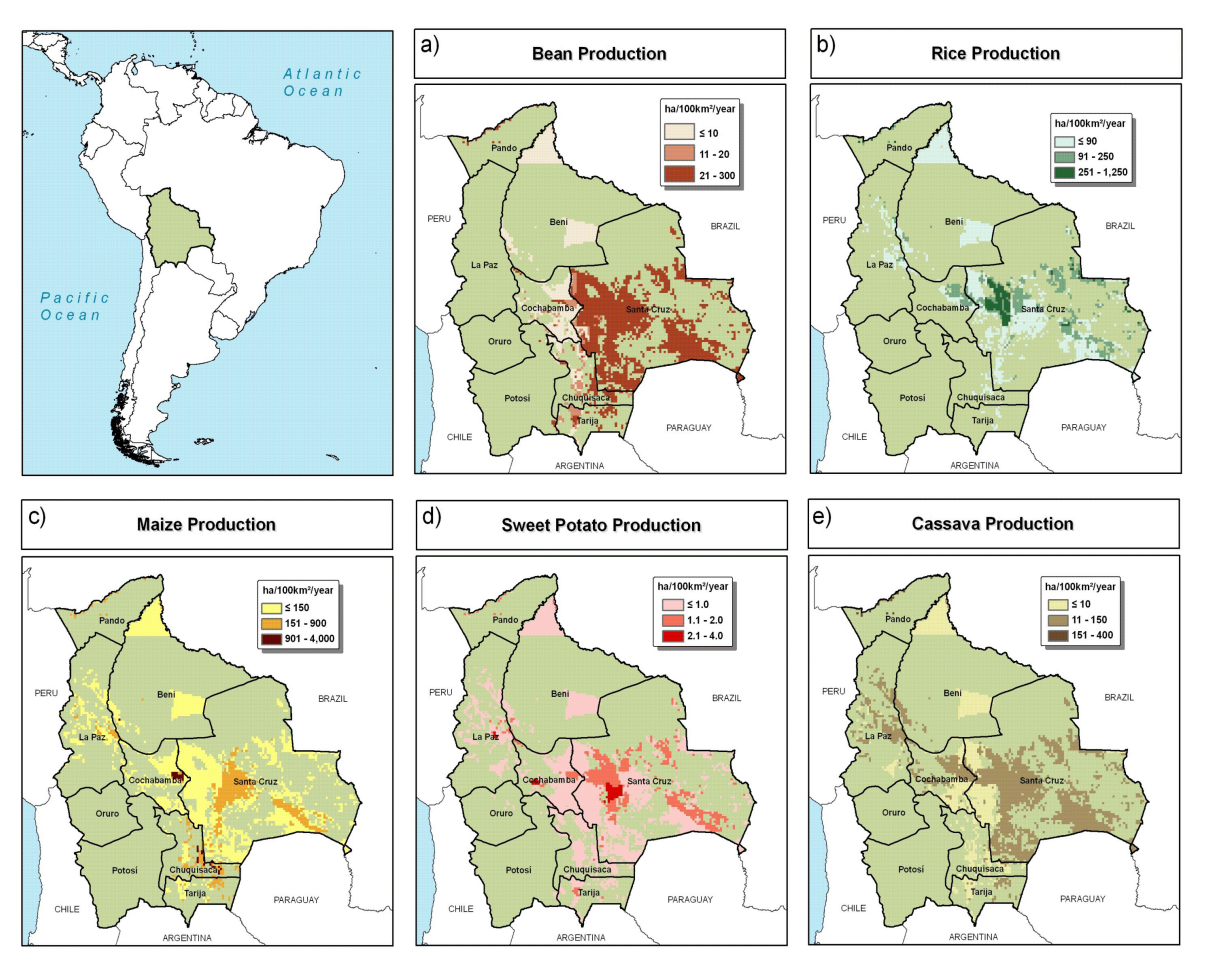
**Crop production in Bolivia**. a) bean, b) rice, c) maize, d) sweet potato, and e) cassava production. Source: You and Wood, 2006 [16].

Maize is the most important crop of those that are the target of biofortification initiatives in Bolivia (Figure 11). Rice and cassava production are important in Santa Cruz department. Bean production is overwhelmingly concentrated in Santa Cruz, with much of it for export [22]. Santa Cruz department is Bolivia's most important in the context of agricultural production.

**Figure 12:**
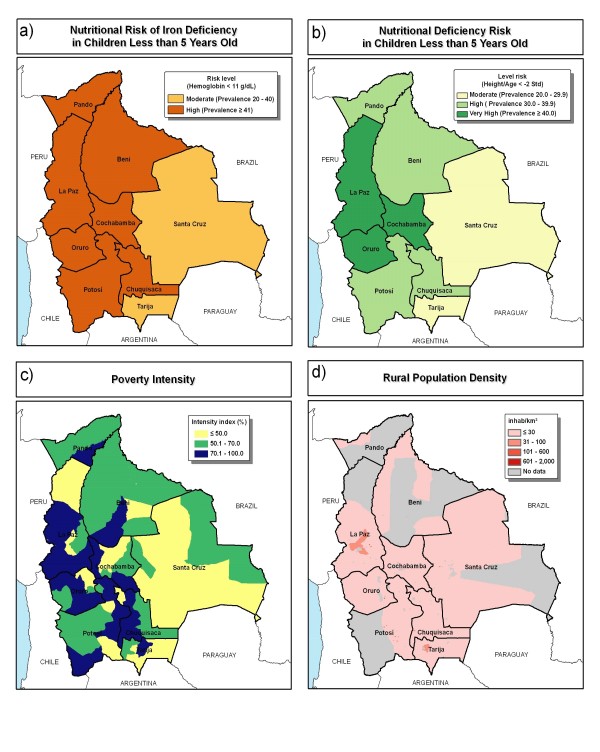
**Variables considered in identifying candidate sites for biofortification in Bolivia**. a) iron deficiency risk and b) height-for-age in children less than 5 years old, c) poverty intensity, and d) rural population density. Sources: MACRO International, 2007 [13] (nutritional deficiency); Schuschny and Gallopin, 2004 [15] (poverty intensity); and CIESIN et al., 2004 [14] (rural population density).

Indicators of risk of nutrient deficiency are moderate, high, or very high throughout Bolivia (Figure 12). Both anemia and stunting indicators suggest the poorest conditions in the western, Andean part of the country. Poverty intensity is higher in the west as well. Crop production and risk of deficiencies do not neatly coincide. While Santa Cruz has comparatively lower risk factors for nutrient deficiencies, its high crop production could make it a focus of biofortification to address nutrient deficiencies, even though they are less severe compared to other countries. The Santa Cruz department could also be the source of biofortified foods for the rest of the country, to the extent that it serves as a breadbasket region.

**Figure 13:**
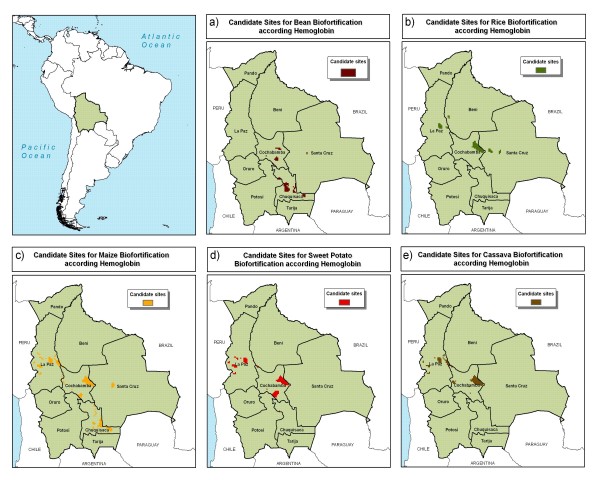
**Candidate sites for iron biofortification in Bolivia**. a) bean, b) rice, c) maize, d) sweet potato, and e) cassava as indicated by hemoglobin levels. Source: AgroSalud, 2007 [8].

**Figure 14:**
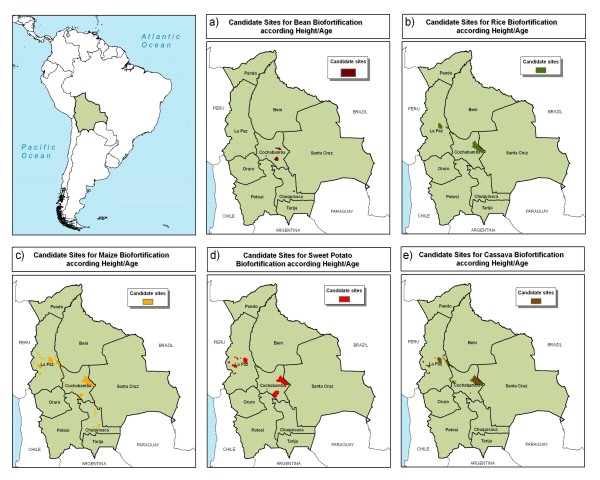
**Candidate sites for zinc, vitamin A, and amino acids biofortification in Bolivia**. a) bean, b) rice, c) maize d) sweet potato, and e) cassava as indicated by height-for-age. Source: AgroSalud, 2007 [8].

Four departments could be strong foci for biofortification in Bolivia – La Paz, Cochabamba, Chuquisaca, and Santa Cruz (Figures 13 and 14). The focus area could extend from the central part of La Paz department towards the southeast near the border with Paraguay. The result maps showed Santa Cruz to be of less interest, mostly due to the relatively lower levels of nutrient deficiency risk. However, Santa Cruz is the most important agricultural region of Bolivia, with good potential for the adoption of biofortified crops.

## Discussion

This study revealed candidate sites for biofortification interventions in Latin America. Data availability, scale problems, and issues specifically related to biofortification need to be addressed to improve the capacity to identify the best sites for disseminating nutrient-dense crop varieties in the region. The following discussion addresses some of these issues.

This research demonstrated that data limitations for geographic targeting of nutrition interventions can be overcome. However, our data collection effort has shown that simply better information could improve geographic targeting of interventions. Very few surveys provide biochemical data on nutrient status. In the three examples described above, two of the countries had hemoglobin data, and none of them had biochemical information indicating risk of zinc, amino acids, or vitamin A deficiency. Anthropometric measures of childhood nutrition are more widely available, but even these can be outdated, depending on the frequency of surveys carried out.

The varying resolution of input data for this geographic targeting exercise reduces its usefulness to some degree. Users of the analysis should be aware of these scale-related problems. The "ecological fallacy" especially limits the analysis when department level data is used [23,24]. The level of nutritional deficiency risk reported for a department may be very high in some parts of the administrative unit and very low in others. For this reason, the results reported here should only be used after consultation with experts who know the situation in a country, or after on-the-ground verification of conditions.

Geographic targeting based on identification of the most severe problem areas is sometimes inappropriate because nutrient deficiency risks may be uniformly severe throughout a country. For example, the level of stunting among children less than 5 years of age is very high in every department of Guatemala [25]. Several other countries only have two categories of deficiency risk. Where nutrition problems are severe everywhere, agricultural considerations such as potential for adoption and level of production should take precedence. In other cases, areas with severe nutritional problems could be served by other interventions aimed at reducing nutrient deficiency, such as supplementation or diet diversification programs. Again, Bolivia provides an example. The department with comparatively less deficiencies – Santa Cruz – may have the greatest potential for biofortification interventions. Even though this department is relatively less poor, moderate nutrient deficiencies are present.

An additional benefit of geographic targeting can be realized by looking for opportunities where more than one crop can be biofortified in a particular region. Programs promoting biofortified crops can realize marginal returns from setting up initiatives for several crops in the same region. These benefits can improve the efficiency of testing biofortified varieties and disseminating them. Ideally, the population of a given place would consume more than one biofortified food. For example, Córdoba department in Colombia could benefit from improved rice, beans, maize, cassava, and sweet potato for supplying diets with higher levels of iron, zinc, tryptophan and lysine (precursors to protein) and vitamin A.

Expert opinion should be used to guide any targeting exercise, thus addressing the data and analysis limitations discussed above. We solicited comments on the results of the weighted overlays from our network of collaborators. There was general agreement about the location of candidate sites for biofortification. Comments tended to focus on contextual conditions for which the analysis could not account. For example, some regions produce crops for export or for animal feed. Others produce crops for urban markets where nutrient deficiency problems may be insubstantial. In other cases, cultural conditions may hinder implementation of biofortification interventions. For example, the people of a region may be accustomed to consuming white-fleshed sweet potatoes – not the high Vitamin A orange-fleshed varieties. Reviews and comments from experts are essential for targeting biofortification interventions.

The data sets and methods described in this paper are oriented towards the current status of information available to conduct a multi-country assessment. Recently, new methods have been applied to create high-resolution nutrition deficiency maps [26]. One such method – called small area estimation – relies on both national censuses and representative household surveys. Using sophisticated statistical analysis, a nutrition risk variable in a household survey, such as height for age, is mapped onto the census geography to create maps at the 2^nd ^administrative level. We are aware of five implementations of this method for mapping malnutrition – in Panama, Dominican Republic, Ecuador, Cambodia, and Bangladesh [27-31]. Until this method is validated and more widely applied, the approach described in this paper provides a low cost alternative for identifying sites for crop biofortification.

## Conclusion

This study demonstrates a method for identifying candidate sites for biofortification interventions. The method uses available secondary data at the finest common spatial resolution currently possible. The study and accompanying data can be used for identifying populations at risk of nutrient deficiencies. It allows designers of large regional nutrition interventions to recognize localities that merit further consideration for inclusion in programs to reduce nutrient deficiency. The research combines agricultural production and health information to support decision-making and program implementation, addressing the need to efficiently target interventions to the populations that need them most.

## Competing interests

EZ, GH, HP, FM, and LV have no competing interests in this study.

## Authors' contributions

EZ developed and pre-processed much of the data, designed and carried out the computer modeling, and wrote the initial manuscript in Spanish. GH led development of the database and contributed to the study design and methodology. HP led the development of nutrition data and their classification as indicators of nutrient deficiency risk. FM developed much of the database and linked statistical information to maps. LV conducted a literature search and developed a table of indicators of the risk of nutrient deficiency. All authors participated in the research design and interpretation of results. They all read and approved the final manuscript.
